# Temporal-Geographical Dispersion of SARS-CoV-2 Spike Glycoprotein Variant Lineages and Their Functional Prediction Using *in Silico* Approach

**DOI:** 10.1128/mBio.02687-21

**Published:** 2021-10-26

**Authors:** Siaw Shi Boon, Chichao Xia, Maggie Haitian Wang, Ka Lai Yip, Ho Yin Luk, Sile Li, Rita W. Y. Ng, Christopher K. C. Lai, Paul Kay Sheung Chan, Zigui Chen

**Affiliations:** a Department of Microbiology, Faculty of Medicine, The Chinese University of Hong Kong, Hong Kong Special Administrative Region, China; b Jockey Club School of Public Health and Primary Care, Faculty of Medicine, The Chinese University of Hong Kong, Hong Kong Special Administrative Region, China; c Stanley Ho Centre for Emerging Infectious Diseases, Faculty of Medicine, The Chinese University of Hong Kong, Hong Kong Special Administrative Region, China; NIH; Harvard Medical School

**Keywords:** COVID-19, SARS-CoV-2, S protein, amino acid variation, *in silico* prediction

## Abstract

SARS-CoV-2 is a positive-sense single-stranded RNA virus with emerging mutations, especially on the Spike glycoprotein (S protein). To delineate the genomic diversity in association with geographic dispersion of SARS-CoV-2 variant lineages, we collected 939,591 complete S protein sequences deposited in the Global Initiative on Sharing All Influenza Data (GISAID) from December 2019 to April 2021. An exponential emergence of S protein variants was observed since October 2020 when the four major variants of concern (VOCs), namely, alpha (α) (B.1.1.7), beta (β) (B.1.351), gamma (γ) (P.1), and delta (δ) (B.1.617), started to circulate in various communities. We found that residues 452, 477, 484, and 501, the 4 key amino acids located in the hACE2 binding domain of S protein, were under positive selection. Through *in silico* protein structure prediction and immunoinformatics tools, we discovered D614G is the key determinant to S protein conformational change, while variations of N439K, T478I, E484K, and N501Y in S1-RBD also had an impact on S protein binding affinity to hACE2 and antigenicity. Finally, we predicted that the yet-to-be-identified hypothetical N439S, T478S, and N501K mutations could confer an even greater binding affinity to hACE2 and evade host immune surveillance more efficiently than the respective native variants. This study documented the evolution of SARS-CoV-2 S protein over the first 16 months of the pandemic and identified several key amino acid changes that are predicted to confer a substantial impact on transmission and immunological recognition. These findings convey crucial information to sequence-based surveillance programs and the design of next-generation vaccines.

## INTRODUCTION

Since the emergence of human coronavirus disease 2019 (COVID-19), which led to the declaration of a pandemic in March 2020, tremendous effort has been invested in researching and battling this deadly ailment (1, 2). The disease was first identified in Wuhan, China, at the end of 2019 and quickly spread across different continents, like Africa, Europe, Oceania, and America, resulting in >150 million confirmed cases in at least 196 countries/regions by the end of April 2021 (https://coronavirus.jhu.edu/map.html). Severe acute respiratory syndrome coronavirus 2 (SARS-CoV-2), the virus that causes COVID-19, is a positive-sense single-stranded RNA virus belonging to the genus *Betacoronavirus* (beta-CoV) (3). The 5′ terminal two-thirds of the genome encodes two large nonstructural polyproteins (open reading frames ORF1a and ORF1b) that further cleaved into a total of 16 nonstructural proteins (NSP1 to NSP16). The discontinuous transcript events generate a nested set of positive-strand virus mRNA for translation into four structural proteins, namely, spike glycoprotein (S), envelope (E), matrix (M), and nucleocapsid (N), as well as six accessory proteins (ORF3a, ORF6, ORF7a, ORF7b, ORF8, and ORF10).

Coronaviruses often undergo genetic recombination, mutation, gain, and loss, which then allow the virus to gain diversification and adaptability to infect mammals, including humans (4–7). Different coronaviruses mutate at different rates. For example, the mutation rate of hCoV-OC43, a beta-CoV that causes the common cold, is approximately 4 × 10^−4^ site/year (8), while SARS-CoV-2 has a relatively higher mutation rate of 1.2 × 10^−3^ to 3.3 × 10^−3^/site/year (9, 10). As of mid-May 2021, SARS-CoV-2 accumulated more than 28,000 single-nucleotide polymorphisms (SNPs) across the whole viral genome (https://bigd.big.ac.cn/ncov/variation/annotation). Among the 4 viral structural proteins, S protein harbors the majority of amino acid variations (11, 12).

The SARS-CoV-2 S protein is a homotrimeric protein involved in host cell attachment, infection, viral entry, and trafficking (13). Each monomer consists of S1 and S2 subunits and a protease cleavage site (amino acid [aa] residue 667/668) located between S1 and S2 (14). S1 contains the N-terminal domain (S1-NTD; aa 14 to 305), receptor binding domain (S1-RBD; aa 319 to 541), hACE2 receptor binding motif (S1-RBM; aa 437 to 508), and C-terminal domain (S1-CTD; aa 542 to 685). S1-RBD plays a pivotal role during the viral infection cycle through binding to the host cell receptors, primarily the human angiotensin converting enzyme 2 (hACE2) (15). In addition, S1-NTD and S1-RBD play a role in manipulating host immune response (16). Meanwhile, S2 is comprised of fusion peptide (FP; aa 788 to 806), heptapeptide repeat sequences (HR1, aa 912 to 984; HR2, aa 1163 to 1213), transmembrane domain (TM; 1214 to 1237), and cytoplasmic domain (CP; aa 1238 to 1273) (17, 18).

Coronavirus S protein is a key target for vaccine and therapy. Compared to other pathogenic coronaviruses, such as severe acute respiratory syndrome coronavirus (SARS-CoV) and Middle East respiratory syndrome coronavirus (MERS-CoV), which emerged in 2002 and 2012, respectively, SARS-CoV-2 S protein binds hACE2 with a higher affinity (18). Accumulated sequencing data on SARS-CoV-2 observed that a key amino acid mutation at position 614, from aspartic acid (D) to glycine (G) in the S protein (S-D614G) in late January 2020, started to spread globally and became dominance beginning in March 2020 (19, 20). Emerging evidence indicates that virus bearing the D614G mutation is associated with faster transmission (21, 22), increased viral infectivity (23, 24), and enhanced viral replication and host fitness (25, 26), which is consistent with higher binding affinity due to a more open conformation toward an hACE2 binding-competent state (27 to 29), suggestive of natural selection on an adaptive benefit. Besides D614G, amino acid changes within S1-RBD, such as N439K, L452R, E484K/Q, and N501Y, also favor virus resistance to monoclonal antibody neutralization (30, 31).

In this study, we focused on S protein mutations that had emerged before the end of April 2021. We outlined the distribution of S protein variations observed among SARS-CoV-2 variant lineages, with a target at four variants of concern (VOCs), including α (B.1.1.7), β (B.1.351), γ (P.1), and δ (B.1.617), that spread quickly worldwide since the end of 2020. Utilizing *in silico* methods, we predicted how amino acid changes in S protein affect their binding affinity to hACE2, major histocompatibility complex (MHC) class I cytotoxic T cell immunogenicity, and B cell epitope probability. We also examined several yet-to-be-identified hypothetical amino acid changes within S1-RBD of VOCs that might confer a lower viral antigenicity and higher binding affinity to hACE2.

## RESULTS

### Global dispersion of SARS-CoV-2.

**FIG 1:**
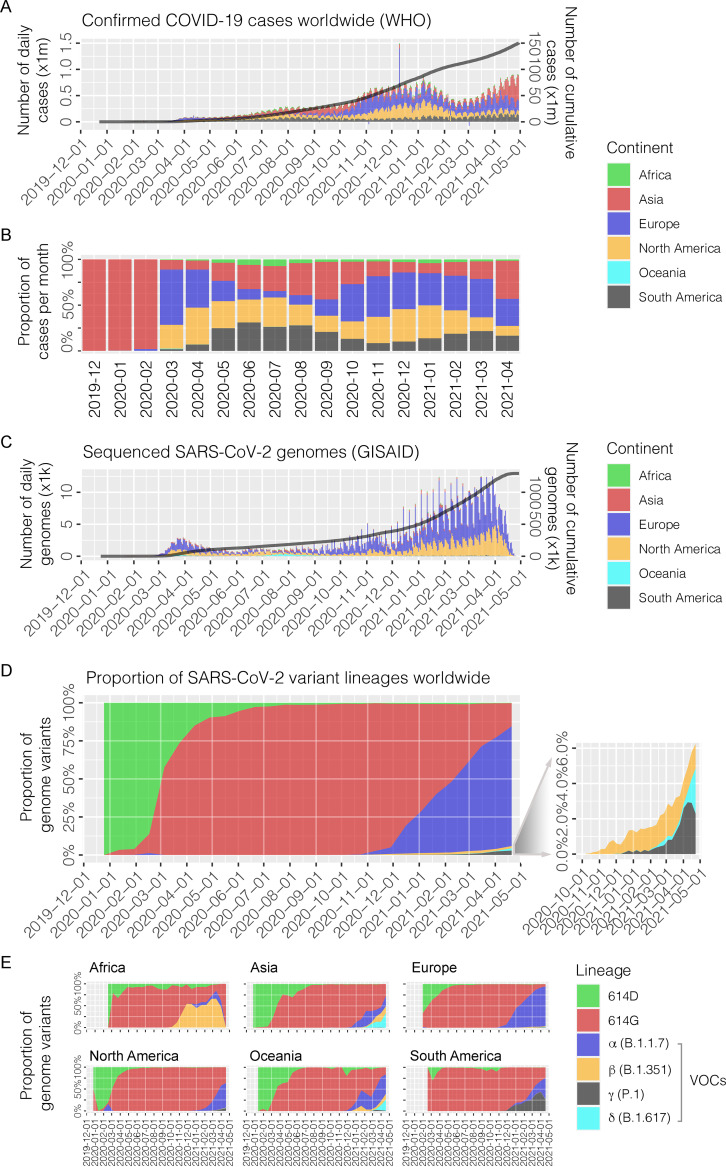
COVID-19 confirmed infections and SARS-CoV-2 complete genomes collected worldwide between December 2019 and April 2021. (A) The number of daily COVID-19 infections reported to the WHO (in millions, x1m). The line in black represents the cumulative number of cases (right *y* axis). (B) The proportion of monthly COVID-19 infections on six continents. Colors in the legend indicate six continents. (C) The number of sequenced SARS-CoV-2 complete genomes available in GISAID according to the date of collection (in thousands, x1k). The line in black represents the cumulative number of genomes. The distribution of cases on six continents is visualized in different colors, as shown in the legend. The proportion of SARS-CoV-2 variant lineages in the world (D) and six continents (E). The main lineages, including the four variants of concern (VOCs), α (B.1.1.7), β (B.1.351), γ (P.1), and δ (B.1.617), are visualized in different colors, as shown. The image on the right highlights three VOCs (β, γ, and δ) with relatively low abundances. 614D, SARS-CoV-2 prototype; 614G, SARS-CoV-2 variant containing glycine at codon position 614 of S gene (S-D614G), except for the four VOCs (α, β, γ, and δ).

Up to 30 April 2021, more than 150 million confirmed cases were recorded worldwide (Fig. 1A). The global spread of SARS-CoV-2 was first recognized in March 2020. Another wave further attacked Europe and North America beginning October 2020, while the proportion of cases increased again in Asia and South America from early 2021 (Fig. 1B). By the end of April 2021, nearly 1.3 million SARS-CoV-2 complete genomes were characterized (Fig. 1C). Compared to the prototype (NC_045512, 614D), numerous nonsynonymous mutations accumulated across the viral genome, with 97.3% of SARS-CoV-2 sequences containing S-D614G in the S protein (Fig. 1D). Among them, 4 S-D614G variants of concern (VOCs), namely, α (B.1.1.7), β (B.1.351), γ (P.1), and δ (B.1.617), emerging with multiple additional amino acid changes in the S1-RBD, have caused community outbreaks since the end of 2020 in different countries (Fig. 1E).

### Genomic diversity of SARS-CoV-2 S protein.

**FIG 2:**
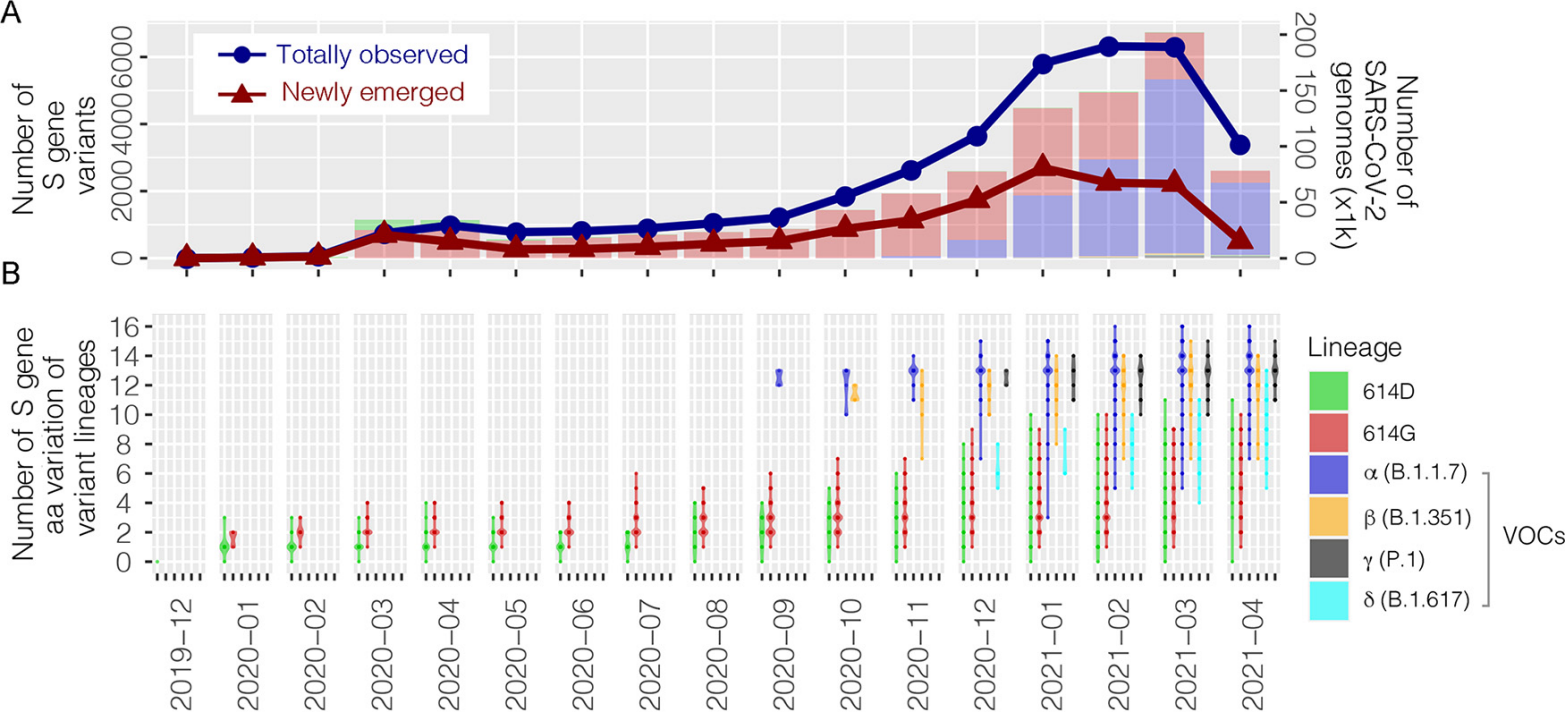
SARS-CoV-2 S gene variants between December 2019 and April 2021. (A) The monthly number of totally observed (blue dots, all detectable unique variants compared to the prototype) and newly emerged S gene variants (red dots, novel unique variants not detected in previous months). The bar chart indicates the number of SARS-CoV-2 genomes each month (right *y* axis, in thousands, x1k), with the distribution of variant lineages visualized in different colors. (B) The monthly number of S gene amino acid (aa) variation of variant lineages. 614D, SARS-CoV-2 prototype; 614G, SARS-CoV-2 variant containing glycine at codon position 614 of the spike (S) gene except for the four VOCs (α, β, γ, and δ). The S gene variants represented by at least 2 identical amino acid sequences were counted.

**FIG 3:**
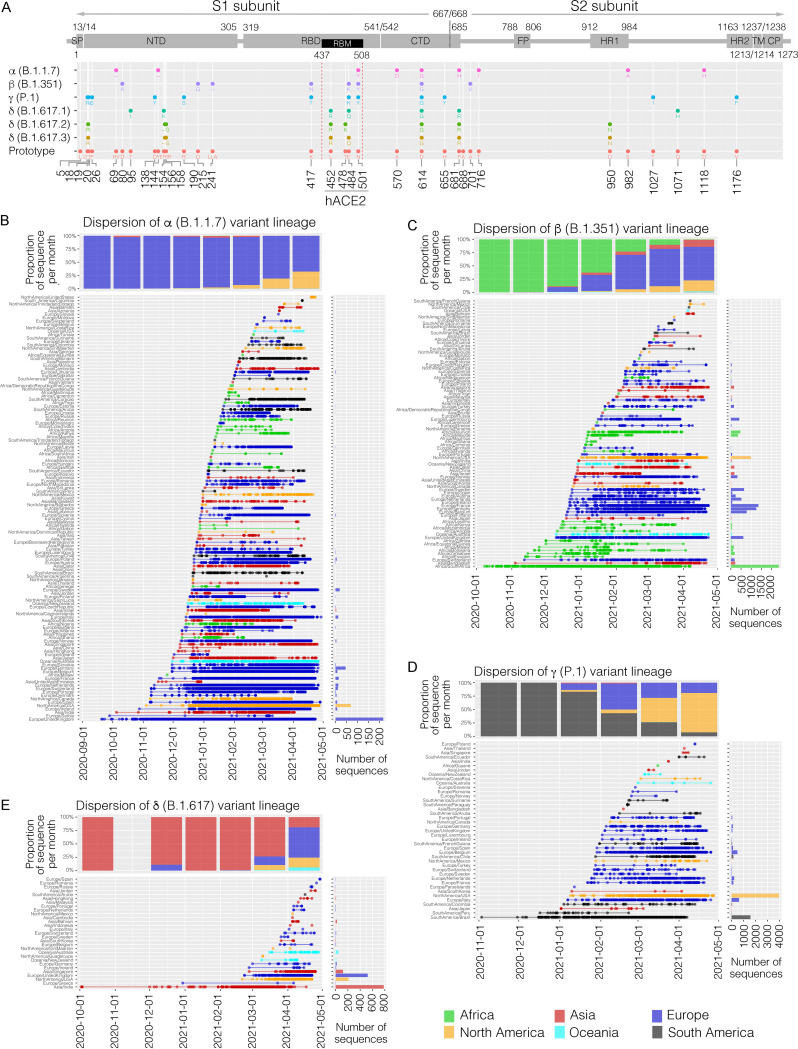
Genetics and dispersion of four variants of concern (VOCs) by the end of April 2021. (A) Conserved amino acid (aa) variations of S protein of four VOCs compared to the prototype (NC_045512). One letter abbreviation of amino acid change is displayed below each dot. The dash (−) represents the deletion event. A schematic representation of SARS-CoV-2 S protein is shown on the top panel. SP, signal peptide; NTD, N-terminal domain; RBD, receptor binding domain; RBM, receptor binding motif; CTD, C-terminal domain; FP, fusion peptide; HR1/2, heptapeptide repeat sequence 1/2; TM, transmembrane domain; CP, cytoplasmic domain. (B to E) Dispersion of SARS-CoV-2 α (B.1.1.7), β (B.1.315), γ (P.1), and δ (B.1.617) variants worldwide, respectively. The dots in each panel indicate a variant sequence of each country reported to GISAID as of the collection date. The bar chart in the top panel represents the proportion of monthly variant sequences between the six continents. The bar chart on the right represents the total number of variant sequences in each country. The colors indicate the six continents that each country belongs to, as shown.

Based on the amino acid sequence alignment of S protein, at least 14,479 unique variants that formed 1,257 Pangolin lineages (defined by Global Initiative on Sharing All Influenza Data [GISAID]) were clustered, with amino acid mutation sites ranging between 1 and 16 (including indel events). Before WHO declared the COVID-19 outbreak a pandemic (December 2019 to February 2020), the S gene was relatively conserved, and 75% (52/69) of unique variants belonged to the prototype (614D) (Fig. 2A), which corresponded to one or two amino acid changes (Fig. 2B). Between March and September 2020, a mean of 430 ± 152 S protein variants emerged per month, with 92% (2,766/3,008) of them containing an S-D614G mutation (614G). Starting from October 2020, when the α (B.1.1.7) variant lineage spread from the United Kingdom, many European countries suffered from a new wave of the pandemic, where there was an exponential emergence of newly observed S protein variants (mean number of 1,815 ± 704 monthly). A proportion of 99% of those novel variants contained the S-D614G mutation together with another 7 ± 5 amino acid variations within the S protein. Of note, there was a trend of increase in the number of the 4 VOCs detected from November 2020 to March 2021, including α (B.1.1.7) (46 < 406 < 947 ∼ 877 < 1173), β (B.1.351) (6 < 17 ∼ 17 < 41 < 49), γ (P.1) (0 < 2 < 14 < 32 < 78), and δ (B.1.617) (0 < 4 ∼ 1 < 18 ∼ 20). The γ (P.1) variant lineage contained 12 consensus mutations with S protein, followed by 9 in α (B.1.1.7), 8 in β (B.1.351), and 7 to 8 in δ (B.1.617) (Fig. 3A). However, it is worth noting that the lower level of mutation identified in the early stage of prepandemic could be due to the limited availability of virus sequences (see the bar chart in Fig. 2A).

The temporal dispersion and reported signature amino acid mutations of the four VOC lineages are described below.

### (i) Alpha lineage, α (B.1.1.7).

The α (B.1.1.7) variant lineage was first recorded in a patient from the United Kingdom in September 2020 (EPI_ISL_601443) (Fig. 3B). Soon after, this variant lineage was found in India in early October 2020 and then in the United States in early November 2020. By the end of April 2021, at least 472,406 α (B.1.1.7) complete genomes were reported from 122 countries/regions across all six continents, with a dramatic increase from December 2020 (3% of total genomes) to April 2021 (76%). Countries like the United Kingdom (*N* = 217,819, 56% of the total sequenced genomes), the United States (70,810, 19%), Germany (46,497, 61%), Sweden (19,126, 63%), and France (14,721, 54%) provided the largest number of sequenced genomes. At least 3,776 S protein variants belonging to α (B.1.1.7) variant lineage were identified, in which all contain 2 deletion events (amino acids 69 to 70 and 144) and 7 mutations (N501Y, A570D, D614G, P681H, T716I, S982A, and D1118H) (Fig. 3A; see also Fig. S1 in the supplemental material). In addition, L5F, L18F, S98F, and K1191N were found in 2.6%, 1.2%, 1.3%, and 2.6% of α (B.1.1.7) S variant sequences, respectively. When aligning the α (B.1.1.7) whole genomes against the prototype, at least 10 conserved amino acid variations within ORF1a (T1001I, A1708D, I2230T), ORF1b (P214L), ORF8 (R52I, Y73C), and N gene (D3L, R203K, G204R, S235F) were observed (Fig. S1).

10.1128/mBio.02687-21.1 FIG S1 Phylogenetic analysis of α (B.1.1.7) S gene variant lineage inferred from the concatenated nucleotide sequence alignment of 12 open reading frames (ORFs). The amino acid variations across the complete genome observed in at least 21 variants were visualized in the heat map. Download FIG S1, TIF file, 0.8 MB. Copyright © 2021 Boon et al. 2021 Boon et al. https://creativecommons.org/licenses/by/4.0/ This content is distributed under the terms of the Creative Commons Attribution 4.0 International license.

### (ii) Beta lineage, β (B.1.351).

The β (B.1.351) variant was first reported from South Africa in October 2020 (EPI_ISL_712081) (Fig. 3C). One month later, it was detected in Bangladesh, Switzerland, and several African countries. Up to April 2021, the β (B.1.351) variant lineage was widely found in many parts of Europe, Asia and North America, with a total of 12,832 sequences reported. Of these, 18% were from South Africa, followed by France (11%), Germany (10%), the United States (8%), Belgium (6%), and other 78 countries/regions across all 6 continents. A total of 139 unique S protein variants of β (B.1.351) were clustered, with the majority carrying 7 consensus amino acid variations (D80A, D215G, K417N, E484K, N501Y, D614G, and A701V) and 1 deletion (amino acid 242) (Fig. 3A, Fig. S2). Fifty-four percent of sequenced β (B.1.351) S genes also contained an L18F mutation. Other than the S protein, the majority of β (B.1.351) genomes had amino acid changes within ORF1a (T265I, K1655N, K3353R), ORF1b (P214L), ORF3a (Q57H, S171L), E (P71L), and N (T205I) (Fig. S2).

10.1128/mBio.02687-21.2 FIG S2 Phylogenetic analysis of β (B.1.315) S gene variant lineage inferred from the concatenated nucleotide sequence alignment of 12 open reading frames (ORFs). The amino acid variations across the complete genome observed in at least 2 variants were visualized in the heat map. Download FIG S2, TIF file, 0.4 MB. Copyright © 2021 Boon et al. 2021 Boon et al. https://creativecommons.org/licenses/by/4.0/ This content is distributed under the terms of the Creative Commons Attribution 4.0 International license.

### (iii) Gamma lineage, γ (P.1).

In early November 2020, Brazil reported a new SARS-CoV-2 variant designated γ (P.1) (EPI_ISL_718682) (Fig. 3D). This variant lineage spread outside South America in early January 2021, and as of April 2021, the United States (*N* = 3,972), Brazil (*N* = 1,580), Italy (*N* = 631), Belgium (*N* = 499), the Netherlands (*N* = 224), and the other 36 countries/regions worldwide have sequenced at least 8,005 γ (P.1) complete genomes, revealing 153 S protein variants. Twelve signature amino acid mutations (L18F, T20N, P26S, D138Y, R190S, K417T, E484K, N501Y, D614G, H655Y, T1027I, and V1176F) were observed in the S protein as well as other consensus changes, including ORF1a (S1188L and K1795Q), ORF1b (P214L and E1164D), ORF3a (S253P), ORF8 (E92K), and N (P80R, R203K, and G204R), across the whole genome (Fig. S3).

10.1128/mBio.02687-21.3 FIG S3 Phylogenetic analysis of γ (P.1) S gene variant lineage inferred from the concatenated nucleotide sequence alignment of 12 open reading frames (ORFs). The amino acid variations across the complete genome observed in at least 2 variants were visualized in the heat map. Download FIG S3, TIF file, 0.4 MB. Copyright © 2021 Boon et al. 2021 Boon et al. https://creativecommons.org/licenses/by/4.0/ This content is distributed under the terms of the Creative Commons Attribution 4.0 International license.

### (iv) Delta lineage, δ (B.1.617).

In early 2021, India faced a new wave of the pandemic. A novel variant lineage was detected within the local community that could be traced back as early as the beginning of October 2020 (EPI_ISL_1415203) (Fig. 3E). This lineage was characterized with a signature mutation, L452R, within S1-RBD. A total of 74 δ (B.1.617) S protein variants revealed from the 1,818 complete genomes were clustered into three sublineages, designated B.1.617.1 (represented by E154K), B.1.617.2 (T19R, T478K), and B.1.617.3 (T19R, T478) (Fig. 3A), which accounted for 62%, 35%, and 3% of sequenced genomes as of the end of April 2021, respectively. It is worth noting that the B.1.617.3 variant sublineage was mainly detected in patients in India (41/51). By the end of April 2021, δ (B.1.617) genomes had been reported from at least 29 countries/regions across 5 continents (except for Africa), mainly in India (*N* = 783), the United Kingdom (*N* = 523), the United States (*N* = 212), Singapore (*N* = 121), and Australia (*N* = 47) (Fig. 3E). Interestingly, δ (B.1.617) variant lineage was highly diversified across the viral genomes; only a couple of consensus amino acid variations were shared by all variants (e.g., P214L in ORF1b; L452R, D614G, and P681R in S protein; V82A in ORF7a; R203M and D377Y in N protein) (Fig. S4). In contrast, a number of amino acid changes were sublineage specific, such as ORF1a-T1567I in B.1.617.1, S-T478K in B.1.617.2, and ORF1a-A2344V in B.1.617.3, suggesting that independent transmissions of δ (B.1.617) ancestors in multiple communities have occurred in the early stage of the outbreak.

10.1128/mBio.02687-21.4 FIG S4 Phylogenetic analysis of δ (B.1.617) S gene variant lineage inferred from the concatenated nucleotide sequence alignment of 12 open reading frames (ORFs). The amino acid variations across the complete genome observed in at least 2 variants were visualized in the heat map. Download FIG S4, TIF file, 0.3 MB. Copyright © 2021 Boon et al. 2021 Boon et al. https://creativecommons.org/licenses/by/4.0/ This content is distributed under the terms of the Creative Commons Attribution 4.0 International license.

### Natural selection of S gene variations.

**FIG 4:**
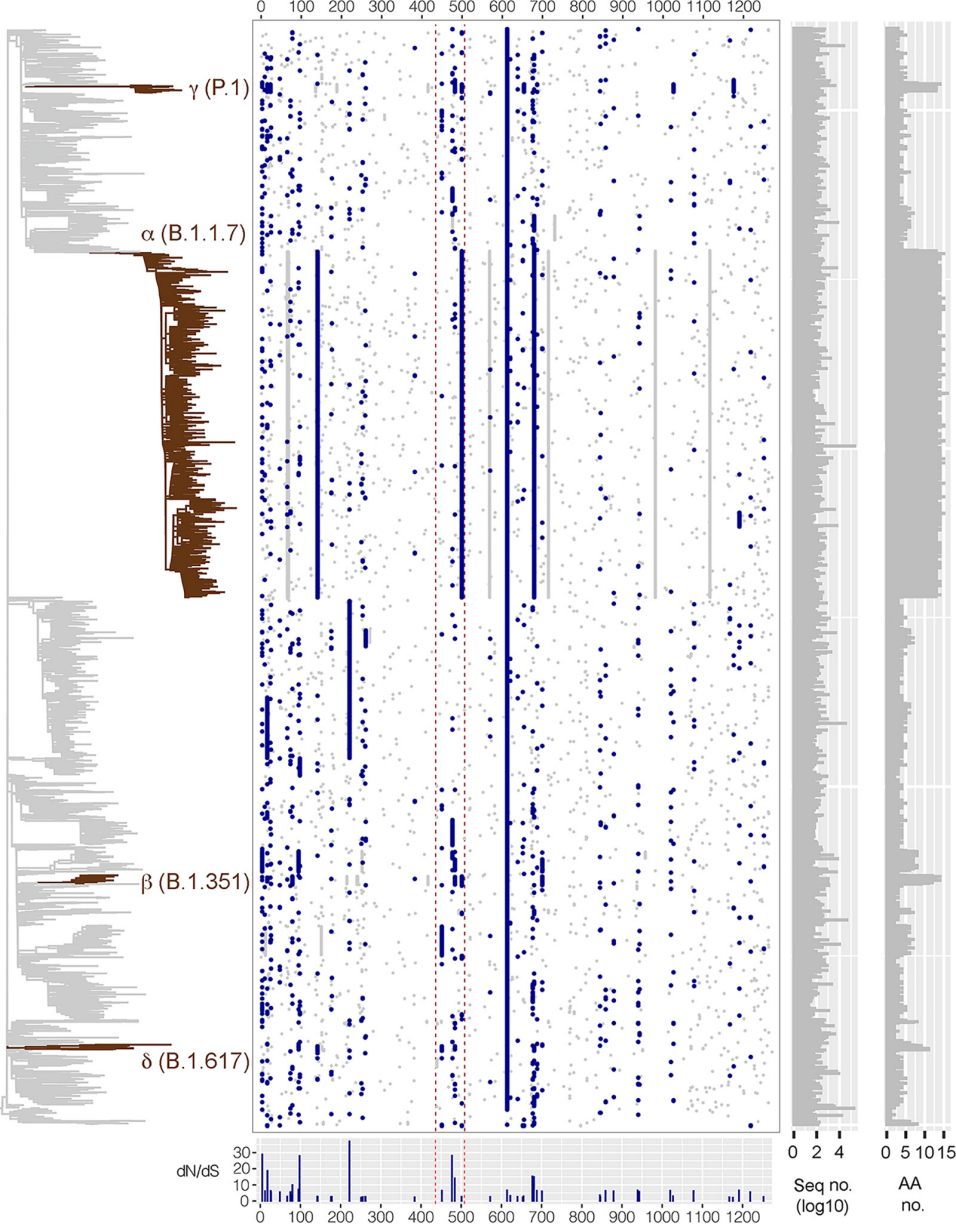
Likelihood ratio tests for positive selection of amino acid (aa) sites for SARS-CoV-2 S gene inferred from 3,242 variants represented by at least 11 identical amino acid sequences. The dots in blue indicate the amino acid sites under positive selection, with *dN*/*dS* ratio using the FUBAR model for natural selection shown in the bottom panel (see amino acid sites and ratio values in Table 1). Other amino acid changes are visualized in gray. The dotted lines in red show S1-NTD and S1-RBM regions of SARS-CoV-2 S gene, of which our amino acid sites that bind to hACE2 were under positive selection (452, 477, 484, and 501). The maximum likelihood tree on the left was constructed using RAxML based on the concatenated nucleotide sequence alignments of 12 open reading frames (ORFs). The phylogenetic positions of the four VOCs (α, β, γ, and δ) are highlighted in dark red. The numbers of sequenced isolates (in log_10_) and amino acid sites of each S gene variant are shown on the right.

When the S protein variants (*N* = 3,242, each represented by at least 11 reported identical sequences) were aligned, at least 693 amino acid variations (including indels) were observed (Fig. 4). Likelihood ratio tests for natural selection showed that at least 51 amino acids in the S protein were under positive selection, including 20 and 5 sites located in the S1-NTD (aa 13 to 305) and the S1-RBD (319 to 541) regions, respectively (Table 1). Among them, 4 amino acid mutations within the S1-RBD (aa 452, 477, 484 and 501) might have an impact on the binding affinity to hACE2. L452R was highly specific to δ (B.1.617) but also found in other variant lineages (e.g., B.1.429). The nonsynonymous change of residue 484 at its first codon position, GAA (glutamic acid, E), could result in AAA (lysine, K), CAA (glutamine, Q) or TAA (stop codon). Interestingly, E484K was commonly observed in β (B.1.351), γ (P.1), P.2, and R.1, and E484Q in δ (B.1.617.1/3). Similarly, changes at residues 501 (N501Y in α, β, and γ; N501T in C.9) and 681 (P681H in α, B.1.1.519; P681R in δ; P618L in B.1.494) under positive selection were also found in several distantly related lineages, suggesting that these mutations have an adaptive benefit in host-virus interaction, probably driven by convergent evolution. Overall, A222V had the highest ratio of nonsynonymous and synonymous site (*dN/dS* ratio) substitutions (FUBAR [fast, unconstrained Bayesian approximation] test, ω=37.06), with a GCT (A) to GTT (V) change at the second codon position commonly observed in B.1.177, followed by residues 5 (ω=28.91), 477 (ω=28.22), and 98 (ω=28.05).

**TABLE 1:** Likelihood ratio tests for the natural selection of 50 amino acids in S protein under positive selection as indicated by the *dN*/*dS* ratio

S gene region	Codon site	FUBAR		SLAC		FEL		MEME		Most common mutation^*a*^	Prevalence of mutation (%) in 4 VOCs^*b*^				Most abundant lineage containing mutation (%)^*c*^
		*dN*/*dS* ratio	*P* value	*dN*/*dS* ratio	*P* value	*dN*/*dS* ratio	*P* value	*dN*/*dS* ratio	*P* value						
											α	β	γ	δ	
SP	5	28.91	0.000	40.15	0.000	49.80	0.000	50.01	0.000	L5F	2.43	3.15	0.45	0.00	B.1.526 (100)
	12	6.14	0.001	9.99	0.000	12.91	0.001	13.05	0.001	S12F	0.19	0.00	1.27	0.00	C.36 (85.71)
NTD	18	18.74	0.000	19.17	0.000	23.17	0.000	23.08	0.000	L18F	1.12	***55.58***	***100.00***	0.00	B.1.177.7 (100)
	26	6.32	0.005	12.50	0.000	14.26	0.003	14.14	0.005	P26S/L/H	0.16	0.44	***100.00***	0.00	B.1.1.372 (51.43)
	27	5.51	0.045	9.50	0.000	13.10	0.031	12.96	0.045	A27S/V	0.11	1.37	0.00	0.00	B.1.356 (26.76)
	49	5.51	0.010	6.28	0.034	11.95	0.006	11.90	0.010	H49Y	0.14	0.00	0.00	0.00	B.1.179 (100)
	67	2.87	0.038	7.00	0.010	9.16	0.026	9.18	0.038	A67V/S	0.20	2.81	0.00	0.00	B.1.1.50 (15.23)
	75	5.60	0.002	8.00	0.002	12.16	0.001	12.02	0.002	G75R/V	0.07	0.00	0.00	0.00	B.1.137 (100)
	76	3.73	0.006	5.50	0.012	9.77	0.004	9.75	0.006	T76I/N	0.21	0.00	0.00	0.00	B.1.1.372 (39.5)
	80	9.93	0.000	7.96	0.013	18.40	0.001	551.83	0.000	D80A/Y	0.03	***100.00***	0.00	0.00	B.1.177.9 (100)
	95	7.22	0.000	15.43	0.000	27.55	0.000	28.05	0.000	T95I	0.22	0.00	0.00	41.22	B.1.526 (100)
	98	28.05	0.000	25.89	0.000	33.59	0.000	33.52	0.000	S98F	1.26	0.44	0.00	0.00	B.1.221 (100)
	142	2.90	0.035	6.49	0.014	9.52	0.023	9.46	0.035	G142V/D/S	0.26	0.00	0.00	***94.45***	B.1.234 (77.71)
	143	2.58	0.007	6.50	0.005	9.22	0.004	9.20	0.007	V143F/D	0.14	0.00	0.00	0.00	B.1.595 (4.96)
	176	2.59	0.004	8.49	0.001	10.39	0.002	10.33	0.004	L176F	0.24	0.41	0.00	0.00	B.1.177.17 (88.93)
	178	2.76	0.000	4.55	0.045	10.02	0.000	21.32	0.000	D178H/N/G	0.23	0.00	0.00	0.00	B.1.36 (23.44)
	222	37.06	0.000	46.50	0.000	64.48	0.000	64.11	0.000	A222V	0.11	0.00	0.00	7.79	B.1.177 (100)
	251	2.23	0.029	5.00	0.017	5.96	0.019	5.99	0.029	P251S/L/H	0.05	0.00	0.00	0.00	AB.1 (100)
	254	2.29	0.018	5.50	0.012	7.09	0.011	7.10	0.018	S254F	0.06	0.00	0.00	0.00	B.1.177.48 (83.62)
	255	2.53	0.024	5.00	0.017	6.44	0.016	6.70	0.024	S255F/Y	0.06	0.00	0.00	0.00	C.30 (100)
	261	2.71	0.004	7.00	0.003	10.62	0.002	10.78	0.004	G261V/R/S	0.06	0.00	0.00	0.00	B.1.1.25 (12.61)
	262	2.44	0.028	4.50	0.026	6.21	0.018	6.24	0.028	A262S	0.03	0.00	0.27	0.00	B.1.177.9 (100)
RBD	384	2.35	0.039	4.50	0.026	5.38	0.026	5.44	0.039	P384L/S	0.18	1.61	0.00	0.00	B.1.463 (100)
	452	6.49	0.000	12.38	0.000	15.59	0.000	15.79	0.000	L452R/Q/M	0.06	0.00	0.00	***100.00***	B.1.429 (100)
	477	28.22	0.000	9.79	0.008	22.62	0.000	22.80	0.000	S477N/R/I	0.06	0.00	0.00	0.00	B.1.526.2 (100)
	484	14.14	0.032	10.28	0.004	30.36	0.021	30.21	0.032	E484K/Q	0.12	***100.00***	***100.00***	***61.43***	P.2 (100)
	501	2.74	0.003	5.74	0.045	16.46	0.002	34.69	0.003	N501Y/T	***99.97***	***100.00***	***100.00***	0.00	C.9 (100)
CTD	572	2.67	0.006	5.50	0.012	9.77	0.004	9.75	0.006	T572I	0.29	0.00	0.00	0.00	B.1.1.486 (100)
	614	6.64	0.011	5.48	0.012	8.36	0.007	8.55	0.011	D614G	***99.99***	***100.00***	***100.00***	***100.00***	B.1.153 (100)
	622	3.31	0.005	7.00	0.003	9.93	0.003	9.97	0.005	V622F/I	0.71	0.00	0.00	0.00	B.1.371 (22.16)
	640	2.70	0.002	9.49	0.000	12.26	0.001	12.17	0.002	S640F	0.16	0.00	0.80	0.00	B.1.177.54 (50.43)
	653	2.29	0.038	4.00	0.039	5.52	0.026	5.51	0.038	A653V	0.19	0.00	0.00	0.00	A.27 (100)
	655	3.02	0.010	6.28	0.033	11.96	0.006	11.99	0.010	H655Y	0.07	0.00	***100.00***	0.00	A.27 (100)
	677	15.31	0.017	11.99	0.036	22.79	0.010	23.30	0.017	Q677H/P/R	0.49	0.00	0.00	0.00	B.1.1.316 (100)
	681	14.97	0.001	10.79	0.000	15.06	0.000	15.44	0.001	P681H/R/L	***99.96***	0.00	0.43	***100.00***	B.1.1.519 (100)
	688	6.42	0.001	10.50	0.000	14.48	0.000	14.35	0.001	A688V/T/S	0.18	0.00	7.83	0.00	B.1.190 (100)
	701	6.06	0.045	9.50	0.000	13.12	0.031	13.21	0.045	A701V/S	0.29	***100.00***	0.00	0.00	B.1.526 (98.89)
	845	3.64	0.017	8.50	0.003	11.16	0.011	11.31	0.017	A845S/V	0.09	2.91	0.55	0.00	B.1.1.317 (94.04)
	846	2.52	0.011	6.00	0.008	8.28	0.006	8.38	0.011	A846V/S	0.03	0.00	0.00	0.00	B.1.1.75 (100)
	859	6.34	0.010	7.49	0.007	13.22	0.006	13.37	0.010	T859I	0.02	0.00	0.00	0.00	B.1.243 (13.71)
	879	6.12	0.012	7.00	0.018	10.00	0.007	10.12	0.012	A879S/V	0.11	0.38	0.00	0.00	B.1.157 (15.79)
HR1	939	6.59	0.001	9.98	0.000	12.90	0.001	13.05	0.001	S939F	0.25	0.00	0.00	0.00	B.1.619 (100)
	943	5.85	0.000	7.11	0.021	14.64	0.002	4,738.16	0.000	S943P/T	0.19	0.00	0.00	0.00	B.1.564 (18.62)
	1020	6.42	0.000	11.50	0.000	15.86	0.000	15.85	0.000	A1020S/V	0.08	0.62	0.00	0.00	B.1.332 (74.45)
	1027	3.04	0.010	4.99	0.018	8.89	0.006	8.85	0.010	T1027I	0.00	0.00	***99.59***	0.00	B.1.619 (100)
	1078	6.26	0.017	8.50	0.003	11.22	0.011	11.20	0.017	A1078S/T/V	0.07	0.00	0.00	0.00	B.1.166 (97.4)
HR2	1167	2.38	0.000	4.00	0.039	6.06	0.020	1,147.30	0.000	G1167V/A	0.01	0.00	0.00	0.00	B.1.1.348 (96.31)
	1176	2.13	0.037	3.99	0.040	5.65	0.025	5.65	0.037	V1176F	0.02	0.00	***100.00***	0.00	P.2 (100)
	1191	6.57	0.002	10.42	0.027	23.94	0.001	24.47	0.002	K1191N	2.53	0.00	0.00	0.00	B.1.177.52 (3.55)
TM	1219	5.59	0.001	10.00	0.000	15.17	0.000	15.38	0.001	G1219C/V	0.24	0.62	0.00	0.00	B.1.350 (100)
CP	1252	2.63	0.000	6.50	0.005	8.36	0.000	8.55	0.000	S1252F/P	0.06	0.00	0.00	0.00	B.1.221 (32.18)

a Observed in at least 100 sequenced genomes.

b Mutations observed in at least 50% of sequenced genomes are in italic and bold type.

c Containing at least 100 sequenced genomes, except for the four VOCs.

### *In silico* structure prediction of S protein variants and classification.

**FIG 5:**
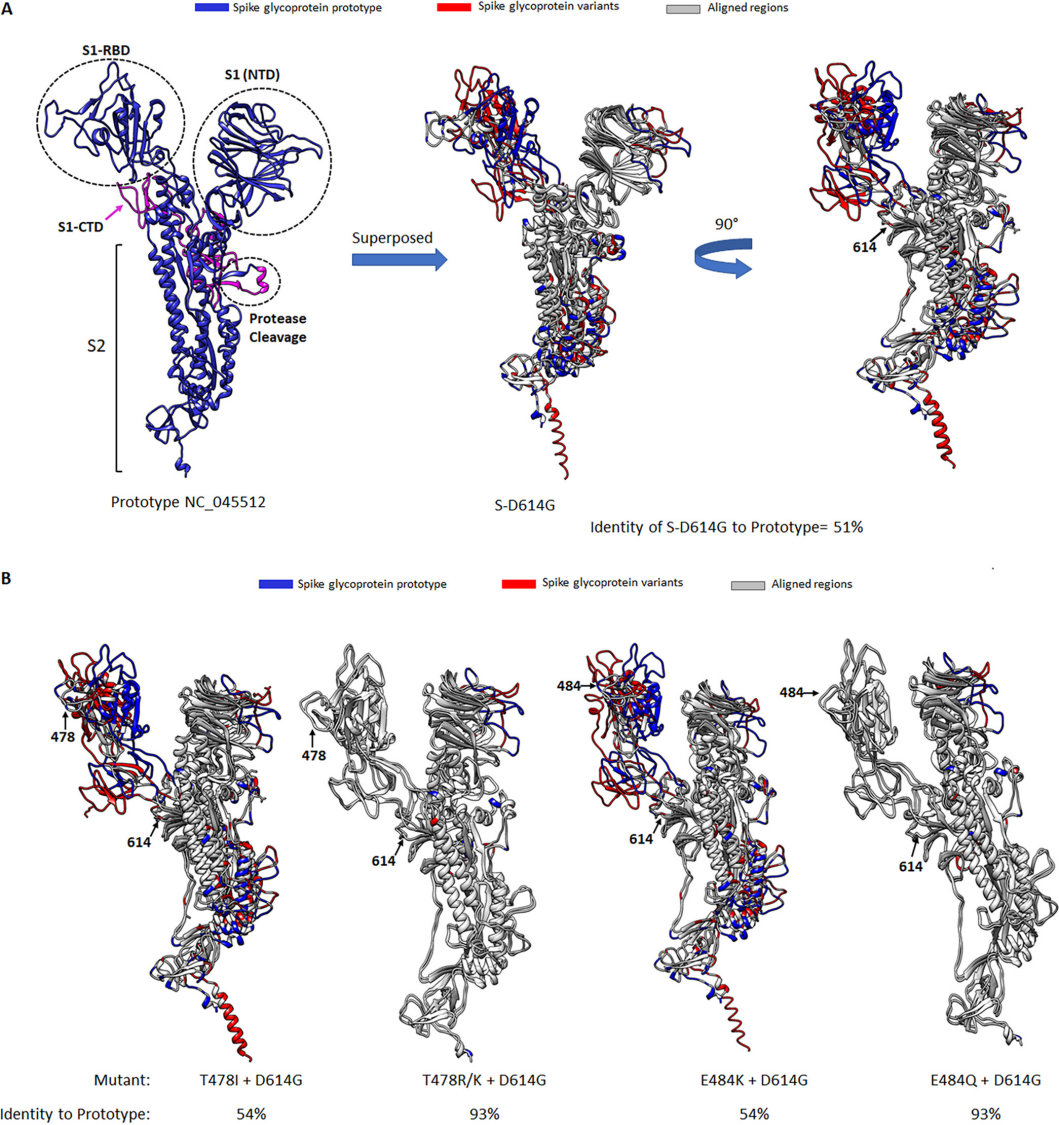
Protein structure homology modeling of SARS-CoV-2 S protein. (A) The protein model of SARS-CoV-2 S protein prototype (NC_045512 in blue) was generated using SWISS-MODEL. The monomer of S protein contains S1 and S2 subunits, with the S1 domain further classified into N-terminal domain (S1-NTD), receptor binding domain (S1-RBD), C-terminal domain (S1-CTD) (colored magenta), and S2 domain. The protease cleavage site is located between S1 and S2. When superposing protein models of S-D614G mutant (mutated amino acid residue 614, indicated by a black arrow) onto the prototype, the aligned regions of the two models are indicated in light gray, while the regions where these two proteins are not aligning are indicated in blue (prototype) and red (S-D614G mutant). Note the D614G mutant is 50.53% identical to the prototype. (B) Protein models of S protein containing T478I/R/K or E484K/Q mutations together with D614G double mutations were superposed onto the prototype. The position of the respective amino acid residues mutated is indicated by black arrows. Note the T478R/K + D614G and E484Q + D614G double mutants are >93% identitical to the prototype, while the S1-RBD domain of T478I + D614G and E484K + D614G double mutants were not aligned with the prototype.

The cryogenic electron microscopy (cyro-EM) structures of S protein for the prototype and α (B.1.1.7) and β (B.1.351) variants are available in the Protein Data Bank (PDB) (27, 32, 33). Utilizing SWISS-MODEL protein homology modeling (34), we predicted structures of >200 variants of interest that were represented by at least 500 different S protein sequences to assess their structural characteristics with reference to the prototype. As S protein is a homotrimeric protein, we superposed monomer using SWISS-MODEL with the closed conformation of S protein (prototype) (PDB entry 6ZGI), generating a predicted structure that was 92% identical to the known structure (Fig. S5). We then superposed the predicted S protein structure of a monomutant containing a D614G mutation. Dual mutants containing D614G and other amino acid changes, including those under positive selection or observed in VOCs (L5F, L18F, S98F, W152L/C, E154K, L222V, and A262S in S1-NTD; N439K, L452Q/R, S477N, L478R/K, E484K/Q, N501Y, and A570D within S1-RBD; Q677H/P and P681H/R in S1-CTD; T716I, S982A, and D1118H in S2) as well as commonly deleted residues (aa 69 to 70, 144, and 241 to 243) were further simulated for structure prediction. From our prediction models, none of the monomutants and deletion mutants affected the S protein structure (>93% identity to the prototype). However, the S-D614G mutation reduced protein structure identity to the prototype by half (51%), altering its S1-RBD conformation primarily (Fig. 5A). When incorporating D614G in monomutants, we were intrigued to find that mutation of T478I + D614G or E484K + D614G resulted in mutants with protein structure like the S-D614G mutant, while other amino acid changes at the same residues, such as T478R/K + D614G or E484Q + D614G, were similar to the prototype (Fig. 5B).

10.1128/mBio.02687-21.5 FIG S5 Superposed S protein predicted using SWISS-MODEL onto cryogenic electron microscopic (cryo-EM) S protein structure deposited in the Protein Data Bank (PDB). The predicted structure of the S prototype (in blue) had 92% identity to the closed conformation of cryo-EM S protein (PDB entry 6ZGI). The predicted structure of the S-D614G mutant (in red) had 97% identity to the down conformation of cryo-EM S-D614G protein (PDB entry 7KRS). Both cryo-EM structures were represented with structures colored in green. Download FIG S5, TIF file, 0.3 MB. Copyright © 2021 Boon et al. 2021 Boon et al. https://creativecommons.org/licenses/by/4.0/ This content is distributed under the terms of the Creative Commons Attribution 4.0 International license.

**FIG 6:**
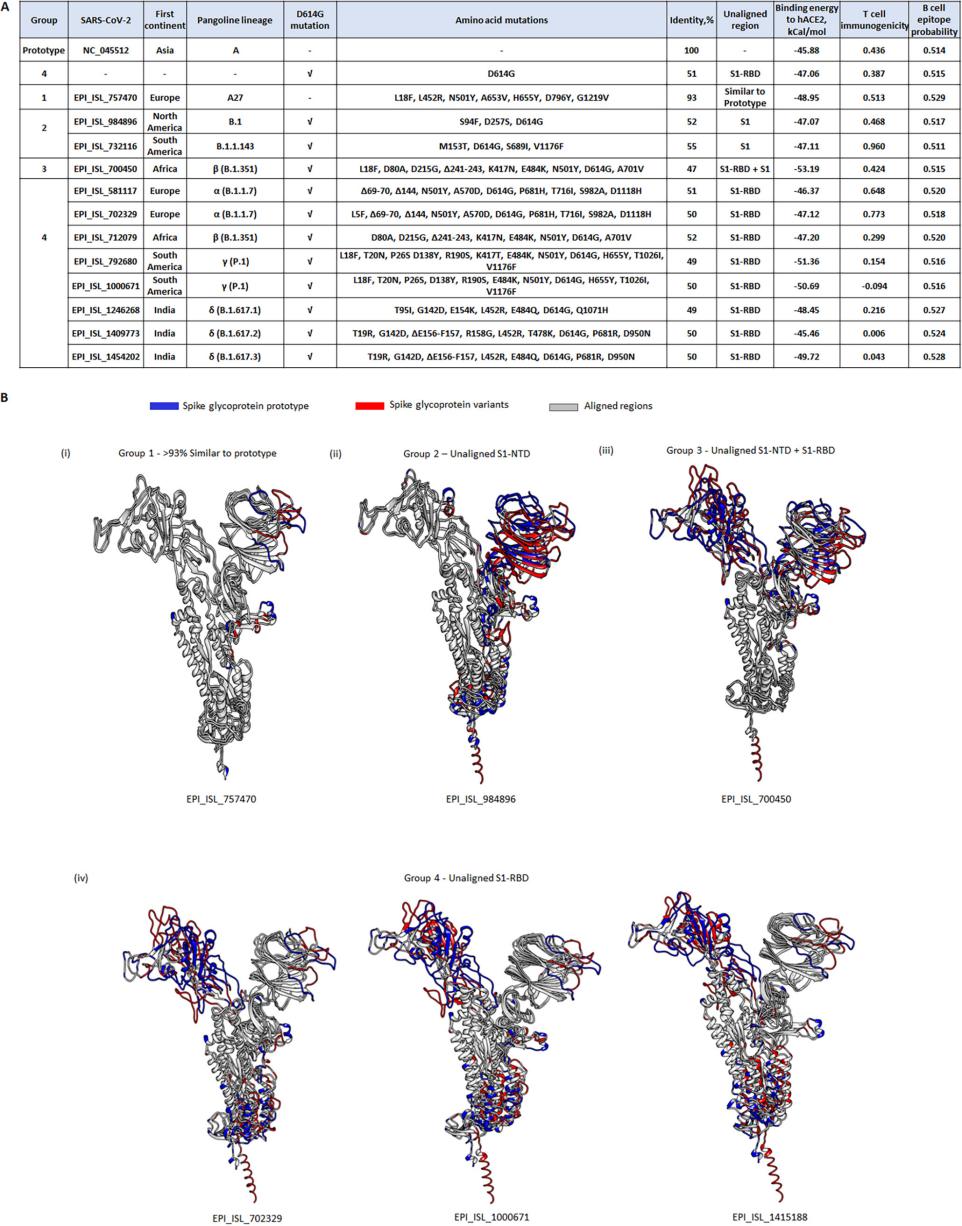
Prediction of S protein structure, binding to hACE2 and antigenicity of SARS-CoV-2 variant lineages. (A) Homology modeling of S proteins of SARS-CoV-2 lineages was performed using SWISS-MODEL. These proteins were classified into groups 1 to 4, based on the region of the protein unaligned with the S prototype. S protein in group 1 did not carry the D614G mutation, while S protein of groups 2, 3, and 4 contained D614G mutation. Also shown are the percent identities of S protein variants to the prototype, predicted binding energy to human angiotensin-converting enzyme 2 (hACE2), major histocompatibility complex class I T cell immunogenicity, and B cell epitope probability of the respective SARS-CoV-2 S protein variant lineages. (B) Protein models of SARS-CoV-2 variants superposed onto the prototype (NC_045512). These proteins were classified into (i) group 1, with the protein sharing >92% structure similarity to the prototype; (ii) group 2, with the S1-NTD of variants unaligned to the prototype; (iii) group 3, with the whole S1 region of S protein variants unaligned to the prototype; and (iv) group 4, with S1-RBD unaligned to the prototype.

Next, we predicted the S protein structure of native variants, including the four VOCs (α, β, γ, and δ), using the same approach. Based on our prediction models, we categorized the variants into 4 groups (Fig. 6A), including group 1 of >92% identity to the prototype (Fig. 6Bi), group 2 of S1-NTD unaligned (Fig. 6Bii), group 3 of S1-NTD and S1-RBD unaligned (Fig. 6Biii), and group 4 of S1-RBD unaligned (Fig. 6Biv). S protein that did not carry D614G mutation was categorized into group 1, while the β (B.1.351) variant that carried additional L18F mutation showed both its S1-NTD and S1-RBD unaligned to the prototype (group 3). As expected, the majority of VOCs carrying mutations within the S1-RBD belonged to group 4.

### Impact of amino acid variations in S protein to hACE2 binding and its antigenicity.

We next utilized *in silico* approaches to assess the impact of S protein conformation divergence on their functions, particularly their hACE2 binding affinity and antigenicity.

**(i) hACE2 binding affinity.** We predicted the binding affinity of S protein variants to hACE2 using pyDock (35). Our results showed that the binding energy of hACE2 with the prototype (NC_045512) and S-D614G mutant was −45.88 kcal/mol and −47.06 kcal/mol, respectively, while all the variants in groups 1 to 4 bound stronger to hACE2 than the prototype. Notably, the S protein binding affinity of the β (B.1.351) variant (EPI_ISL_700450 in group 3) bound the strongest to hACE2 (−53.19 kcal/mol), followed by γ (P.1) (−51.36 kcal/mol), δ (B.1.617.1 and B.1.617.3) (−48.45 and −49.72 kcal/mol, respectively), α (B.1.1.7) (−47.12 kcal/mol), and δ (B.1.617.2) (−45.46 kCal/mol) variant lineages (Fig. 6A). In addition, our results showed that β (B.1.351) and α (B.1.1.7) variants bound stronger than S-D614G to hACE2, which is consistent with previous reports based on both *in silico* and *in vitro* studies (36, 37).

**(ii) Antigenicity of S protein variants.** As S protein is known to elicit host immune response, we used immunoinformatics tools to predict the antigenicity of the S protein prototype and variants. We used Immune Epitope Database (IEDB) analysis resources to predict the ability of S protein sampled by MHC class I molecules to be presented as antigens. BepiPrep-2.0 (38) was used to predict the probability of S protein being recognized by B cells to produce S-specific immunoglobulins. Our results showed that S protein variants in groups 1, 2, 3, and α (B.1.1.7) in group 4 had a greater, while β (B.1.351), γ (P.1), and δ (B.1.617) variants in group 4 had a lower, T cell immunogenicity than the prototype (Fig. 6A). For B cell epitope probability, S proteins featured in groups 1, 2, and 3 scored higher, except for the B.1 variant lineage (EPI_ISL_984896, group 2), which scored similar or higher than the prototype and D614G mutant. S proteins featured in group 4 had a lower B cell epitope probability than the prototype and D614G mutant. These findings indicated that mutation harbored within the VOCs conferred a lower T and B cell immunogenicity of S proteins, allowing the virus to circumvent host immune surveillance.

### Prediction on potential amino acid alterations that confer lower immunogenicity.

As SARS-CoV-2 continues to mutate, we attempted to predict amino acid mutations that could emerge in SARS-CoV-2 variants. Based on *dN/dS* ratio and key amino acids involved in immune evasion, we focused on L5, L18, S98, and A222 (S1-NTD), N439, L452, S477, E484, and N501 (S1-RBD), and Q677 and P681 (S1-CTD). We changed each of the nucleotides corresponding to these genetic codes and predicted the potential amino acid mutation that could arise, as shown in Table 1 and Table S1. Subsequently, using the prototype as a template (NC_045512), we artificially replaced the respective native S protein variants with the predicted amino acids. As D614G mutation achieved a stable mutation, we incorporated the artificial and D614G mutations and analyzed the impact of these amino acid changes to (i) S protein structure and (ii) T and B cell antigenicity.

10.1128/mBio.02687-21.6 TABLE S1 Summary of S protein structure homology, frequently mutated key amino acid sites, prediction of potential mutation of these key amino acid sites, prediction of their major histocompatibility complex class I T cell immunogenicity, and B cell epitope probability. Download Table S1, XLSX file, 0.02 MB. Copyright © 2021 Boon et al. 2021 Boon et al. https://creativecommons.org/licenses/by/4.0/ This content is distributed under the terms of the Creative Commons Attribution 4.0 International license.

**(i) Impact of artificial amino acid change on S protein structure.** Our S protein structure models revealed that all artificial D614G dual mutations containing amino acid changes in S1-NTD, S1-RBD, S1-CTD, or S2 shared a structure that resembled the S-D614G mutant. As mentioned, an exception was observed with T478R/K + D614G or E484Q + D614G, in which these mutants resembled the prototype (Fig. 5B).

**(ii) Prediction of artificial amino acid change to T and B cell recognition.** The prototype had an overall T cell immunogenicity and B cell epitope probability score of 0.436 and 0.470, respectively, while the overall T and B cell immunogenicity scores of the D614G mutant were 0.387 and 0.470, respectively. Among the amino acid mutations found in circulating SARS-CoV-2 variants, E484Q/K, P681H/R/L/S, P936Y/N/H, D950H/A/N, and D1118H/Y had a lower T cell immunogenicity than the prototype and S-D614G mutant (Table S1). Among the artificial mutations, A222P/S, N439S, L452P, T478P/S/N, E484L/A/G/V/D, N501S/K, Q677K, P681A/T, T716S/P/K, P936Q, D950Y/Q, and D1118Q resulted in S protein variants with a lower T cell immunogenicity than the prototype and D614G mutant (Table 2).

**TABLE 2:** The predicted artificial amino acid changes potentially confer to lower T cell immunogenicity and B cell probability of Spike glycoprotein

Native amino acid	Amino acid change	T cell immunogenicity score	B cell epitope probability (entire S protein)
Prototype		0.436	0.470
D614	G614	0.387	0.470
A222	P222	0.386	0.467
	S222	0.236	0.468
N439	S439	0.280	0.468
L452	P425	0.387	0.468
T478	S478	0.188	0.469
E484	G484	0.322	0.467
	V484	0.329	0.467
	D484	0.311	0.467
N501	K501	0.183	0.467
Q677	K677	0.338	0.469
T716	S716	0.188	0.468
	P716	0.338	0.467
	K716	0.139	0.469
P936	Q936	0.252	0.468
D950	Y950	0.361	0.467
	Q950	0.252	0.468
D1118	Q1118	0.252	0.468

To understand the likeliness of artificial amino acid in altering B cell epitope probability, we assessed the effect of amino acid alterations on the overall B cell epitope probability of the entire S protein. S1 that contained L5V/H/P/R, L18V/I/H/R/P, S98/A/P/Y/C, A222P/T/S/G/D, K417E/Q/R/G/M, N439Y/I/S/T, T478A/S, E484G/V/D, N501I/E/D/K, T716S/P/A/R/K, D936R/A/Q, and D950R/Y/V/G/Q, S982T/P/L, and D1118R/G/A/Q artificial mutations resulted in S proteins with a lower B cell recognition ability than the respective native amino acids (Table 2, Table S1). Altogether, our findings indicate that mutations on A222, N439, L452, T478, E484, N501, Q677, T716, D936, D950, and D1118 impact virus T and B cell immunogenicity. Notably, we predicted that the presence of N493S, T478S, E484D, N501K, T716K, and D950Q mutations in SARS-CoV-2 variants could confer lower immunogenicity than the native variants.

### Predict the impact of artificial amino acid mutations on S protein functions of SARS-CoV-2 variants.

We assessed the impact of artificial mutations on S protein functions of the 4 VOCs (α, β, γ, and δ). We focused on artificial mutations in S1-RBD (N439S, L452P, S477G, T478S, E484D, and N501K) that resulted in S protein conferring a lower antigenicity (Table 2) and predicted their protein conformation, hACE2 binding affinity, and antigenicity. These results were summarized in Table 3.

**TABLE 3:** Potential impact of artificial mutation on protein conformation, T cell immunogenicity, B cell epitope probability, and binding affinity to hACE2 of Spike glycoproteins of VOCs

SARS-CoV-2	First continent	WHO label (pangolin lineage)	Amino acid change	Identity, %	Group	T cell immunogenicity	B cell epitope probability (S1-RBD)	hACE2 binding energy, kcal/mol
NC_045512	Asia			100	Prototype	0.436	0.512	−45.88
NC_045513	Asia		D614G	50.53	4	0.387	0.515	−47.06
EPI_ISL_702329	Europe	α (B.1.1.7)		50.44	4	0.773	0.518	−47.12
			N439S	48.50	4	0.618	0.531	−49.08
			L452P	53.27	4	0.773	0.537	−48.99
			S477G	53.27	4	0.967	0.514	−46.09
			T478S	48.50	4	0.574	0.523	−47.96
			E484D	48.41	4	0.697	0.518	−49.11
			N501K	48.50	4	0.566	0.519	−49.01
EPI_ISL_700450	Africa	β (B.1.351)		46.91	3	0.424	0.515	−53.19
			N439S	53.98	3→4	0.271	0.513	−53.46
			L452P	48.32	3	0.424	0.526	−51.72
			S477G	48.32	3	0.618	0.522	−46.64
			T478S	53.98	3→4	0.225	0.513	−53.33
			E484D	54.77	3→4	0.655	0.516	−48.17
			N501K	49.29	3	0.217	0.512	−53.36
EPI_ISL_792680	South America	γ (P.1)		48.85	4	0.154	0.516	−51.36
			N439S	48.76	4	−0.001	0.523	−52.13
			L452P	53.18	4	0.154	0.531	−52.01
			S477G	53.18	4	0.348	0.518	−48.89
			T478S	48.76	4	−0.045	0.515	−45.22
			E484D	48.76	4	0.385	0.515	−48.97
			N501K	48.76	4	−0.053	0.508	−49.02
EPI_ISL_1246268	India	δ (B.1.617.1)		48.67	4	0.216	0.527	−48.45
			N439S	48.59	4	0.061	0.531	−58.89
			L452P	48.59	4	0.155	0.529	−48.20
			S477G	48.67	4	0.410	0.522	−59.42
			T478S	48.59	4	0.017	0.524	−48.60
			E484D	48.59	4	0.350	0.522	−54.81
			N501K	48.59	4	0.012	0.529	−48.80
EPI_ISL_1409773	India	δ (B.1.617.2)		50.27	4	0.006	0.524	−45.46
			N439S	48.50	4	−0.149	0.529	−46.77
			L452P	48.50	4	0.006	0.526	−46.89
			S477G	48.67	4	0.200	0.524	−50.33
			T478S	48.50	4	0.054	0.525	−47.87
			E488D	48.41	4	−0.070	0.523	−48.96
			N501K	48.50	4	−0.198	0.521	−47.42
EPI_ISL_1454202	India	δ (B.1.617.3)		50.27	4	0.043	0.528	−49.72
			N439S	48.23	4	−0.112	0.532	−53.83
			L452P	48.32	4	−0.018	0.527	−49.21
			S477G	48.41	4	0.237	0.520	−49.74
			T478S	48.23	4	−0.156	0.523	−53.96
			E488D	48.23	4	0.177	0.527	−47.79
			N501K	48.23	4	−0.161	0.529	−47.17

**(i) S protein conformation.** We superposed S protein models of α (B.1.1.7), β (B.1.351), γ (P.1), and δ (B.1.617) containing artificial amino acid mutations against the prototype and S-D614G mutant. In general, there was no change in the conformation of S proteins, except for β (B.1.351) variants represented by EPI_ISL_700450 (group 3) and EPI_ISL_712079 (group 4) (Fig. 6A and B). Strikingly, when N439S or T478S was simulated, or E484K mutation was replaced with E484D in the amino acid sequence of EPI_ISL_700450, its S protein structure featured in group 3 switched to group 4 (Table 3). On the contrary, adding N439S and L452P or mutations into the existing EPI_ISL_712079 S protein led to a switch of structure featured in group 4 to group 3 (Table S2).

10.1128/mBio.02687-21.7 TABLE S2 Prediction of the presence of predicted artificial amino acid mutation in SARS-CoV-2 S protein variant lineages, and the impact on their protein residue similarity, root-mean-square-deviation (RMSD), protein structure grouping, major histocompatibility complex class I T cell immunogenicity, B cell epitope probability, and binding energy to human angiotensin converting enzyme 2 (hACE2). Download Table S2, XLSX file, 0.02 MB. Copyright © 2021 Boon et al. 2021 Boon et al. https://creativecommons.org/licenses/by/4.0/ This content is distributed under the terms of the Creative Commons Attribution 4.0 International license.

**(ii) hACE2 binding affinity.** Artificially mutated S proteins of the SARS-CoV-2 variant lineages displayed a different impact on hACE2 binding affinity. For example, insertion of N439S, L452P, T478S, E484D, or N501K mutations into the existing α (B.1.1.7) S protein conferred a higher binding affinity to hACE2. Similarly, S proteins of β (B.1.351) variants with N439S or T478S insertion, or alteration of N501K to N501Y, resulted in a higher binding affinity to hACE2. Meanwhile, incorporation of N439S artificial mutation into the existing mutations of γ (P.1) and δ (B.1.617) also resulted in increased binding affinity to hACE2 than their respective natural mutants.

**(iii) T and B cell antigenicity.** Since the artificial mutations cluster within the S1-RBD, we further examined the B cell epitope probability of the S1-RBD among the variants. The presence of N439S, T478S, and N501K artificial mutations in all four VOCs led to a lower T cell immunogenicity than the respective natural variants. On the other hand, the presence of S477G in α (B.1.1.7), N439S, T478S, and N501K in β (B.1.351), T478S, E484D, and N501K in γ (P.1), and E484D in δ (B.1.617) resulted in S protein variants with a lower B cell epitope probability.

Collectively, our prediction models indicate that three yet-to-be-identified hypothetical mutations (N439S, T478S, and N501K) alter S protein structure coupled with increased binding affinity to hACE2 and lower immunogenicity. These mutations may confer additional adaptive and survival benefits to the current circulating SARS-CoV-2 variants.

## DISCUSSION

With the ramping up in sequencing capacity and interest, an enormous number of novel variants of SARS-CoV-2 were identified. Mutation occurs partly due to natural selection (39), and the mutation rate reflects a response to the selection (40). The mutation rate of SARS-CoV-2 is higher than that of the mild beta-CoVs, such as hCoV-OC43 (8), but lower than that of RNA viruses in general (41).

In this study, we focused on mutations in the S protein, particularly the key amino acids under positive selection pressure. After stringent filtering, we identified 693 amino acid variations in S protein. Among these amino acid mutations, S-D614G is the mutation that emerged in January 2020 (20, 23) that allows SARS-CoV-2 to bind to cell receptors, predominantly hACE2, and enter host cells more easily (25). Since the pandemic was declared, strict travel restrictions have been imposed in most countries. Despite this, S-D614G continued to be detected in different countries, indicating that SARS-CoV-2 S-D614G mutant was the parental virus seeding across continents and then continued to mutate independently, giving rise to SARS-CoV-2 variants containing additional signature amino acid mutations.

The amino acid mutation may confer protein structural and functional alteration. The structural change may depend upon the change in the group of charge, polarity, and hydrophobicity of the amino acid and whether or not the amino acid alteration occurs within the functional domain of the protein. For S protein, our prediction models and others (28) showed that single-amino-acid mutation alone can alter the conformation of S1-RBD. Amino acid change of T478I (polar to nonpolar residue) and E484K (negative- to positive-charge residue) did not contribute to the major shift of the S-D614G structure. However, T478R/K (polar to positive-charge residue) and E484Q (negative to polar residue) resulted in a shift of S-D614G to a structure resembling that of the prototype, while amino acid change can lead to a change in S protein antigenicity and binding affinity to hACE2. Of note, the nonsynonymous substitution of frequently mutated amino acids influences S protein conformation. For instance, the B.1.351 variants with an additional L18F mutation resulted in altered S1-NTD and S1-RBD conformation with increased binding affinity to hACE2.

N501Y has also been shown to confer increased infectivity. Even though both the B.1.1.7 and B.1.351 variants harbor the N501Y mutation, they display a different degree of infectivity and antibody neutralization ability. The B.1.351 variant possesses greater infectivity and better resistance to antibody neutralization (42). In addition, other amino acids are also important to viral pathogenicity. SARS-CoV-2 K417T/E484K dual mutants resist postvaccination serum neutralization to an extent similar to that of the B.1.351 variant (42), while N439K mutation arose independently in different countries, facilitating viral attachment to hACE2 and resistance to antibody neutralization (43). Aside from the known mutations, we predicted that if SARS-CoV-2 acquires N439S, T478S, or N501K mutations, the virus will attain a chance to infect host cells even more efficiently and with reduced antigenicity.

Mutation via amino acid deletion may affect virus fitness and is known to influence a wide variety of biological phenomena. However, this may depend on whether the deletion occurs in the vicinity of a functional domain. From our predictive models, deletion of amino acids 69 to 70 and 144, which are signatures of B.1.1.7 variants, may enhance the infectivity of the virus; however, this may not affect its immunogenicity, while deletion of amino acids 241 to 243, located relatively closer to S1-RBD, found in B.1.351, renders lower immunogenicity and higher binding affinity to hACE2.

However, there are several shortfalls of our *in silico* prediction models. First, S protein models were generated based on the protein structure deposited in the PDB, and amino acid mutations predicted have not been reported before. The precise protein structure of novel mutants might be inaccurate. Second, the docking model utilized a rigid protein structure to predict binding energy, which differs from the natural status of activated S protein when encountering hACE2 or antagonist antibodies (44). Finally, despite the immunoinformatics tools being useful to predict the antigenic epitope of a protein, they are far from perfect. Therefore, laboratory evidence is paramount to further elucidate the functionality and antigenicity of S protein variants. Nonetheless, our findings provided a foundation to further investigate how SARS-CoV-2 exhibits its infectivity and pathogenicity. Despite these deficiencies, our findings are impactful for preparing the potential threat posed by novel S protein mutations in relation to viral pathogenicity. More importantly, our data are of great importance particularly for vaccine development and anti-SARS-CoV-2 drug discovery.

## MATERIALS AND METHODS

### Metadata and sequence collection.

We retrieved 1,294,693 high-quality SARS-CoV-2 complete genome sequences deposited in the GISAID database, with metadata available from 24 December 2019 to 30 April 2021. Among these, 939,591 amino acid sequences encoding the complete S protein were extracted and clustered into 34,134 unique variants using cd-hit (*-G 1 -aL 0.0 -c 1 -M 0 -T 0 -d 0 -g 1 -sc 0*) (45). For each variant, the isolate with the earliest collection date was selected as the representative sequence. After removing singletons (*N* = 19,655), 14,479 variants represented by at least 2 identical S amino acid sequences were retained for further analyses.

### Phylogenetic analysis.

To assess the evolutionary relationship of the major circulating SARS-CoV-2 variant lineages, the concatenated 12 gene (1a, 1b, S, 3a, E, M, 6, 7a, 7b, 8, N, and 10) nucleotide sequences inferred from the S protein variants (*N *= 3,242) were aligned using MAFFT v7.402 (46). The maximal likelihood (ML) tree based on these aligned genomes was constructed using RAxML MPI v8.2.12 (47) with optimized parameters via CIPRES Science Gateway (48).

### Detection of positive selection.

Four algorithms within the HyPhy distribution (49), including FEL (fixed-effects likelihood), FUBAR (fast, unconstrained Bayesian approximation), MEME (mixed-effects model of evolution), and SLAC (single-likelihood ancestor counting), were used to detect the sites under positive selection. An ML tree inferred from 3,242 S gene variant complete genome nucleotide sequences was preconstructed as the phylogenetic input. A codon site with a *dN*/*dS* (ω) value of >1 was regarded as under positive selection when the posterior probabilities inferred from the four algorithms were ≥0.95 (*P ≤ *0.05).

### Homology modeling of protein structure, structure comparison, and function prediction.

S protein structures were predicted using the SWISS-MODEL (34), an automated homology modeling approach, based on SARS-CoV-2 S variant amino acid sequences available in the Protein Data Bank (PDB) (PDB entries 6ZP2, 6VXX, 7LSS, 7LYQ, 7CWN, 7CN8, and 6LXT). Molecular docking of SARS-CoV-2 S proteins and hACE2 (residues 23 to 63) was performed using pyDock (35) and Chimera UCSF (50). The percent identity of S protein variants and prototype was analyzed using Chimera UCSF. The total binding energy of S proteins to hACE2 was predicted using pyDock. Prediction on major histocompatibility complex (MHC) class I T cell immunogenicity was performed using IEDB analysis resources (51, 52), while B cell epitope probability of the S1-RBD or entire S protein was predicted using BepiPrep-2.0 (38).

### Data availability.

The complete genome sequences of coronaviruses analyzed in this study were downloaded from the Global Initiative on Sharing All Influenza Data (GISAID).
