# Supplementary figures and images for: Temporal-Geographical Dispersion of SARS-CoV-2 Spike Glycoprotein Variant Lineages and Their Functional Prediction Using in Silico Approach
Source: mBio. 2021 Oct 26;12(5):e02687-21. doi: 10.1128/mBio.02687-21 (PMC8546546; doi:10.1128/mBio.02687-21)

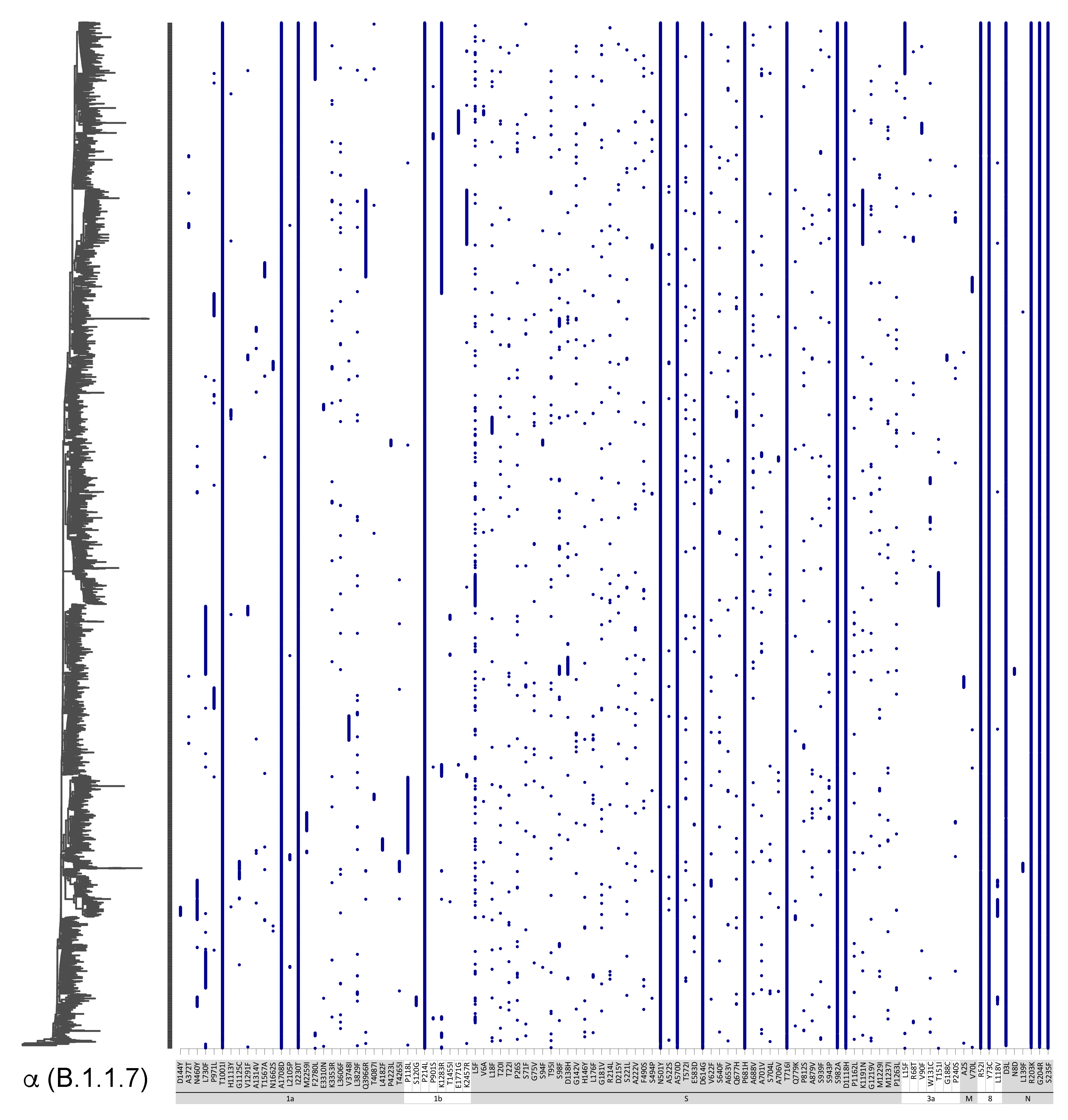

Supplement: FIG S1 [file mbio.02687-21-sf001.tif]

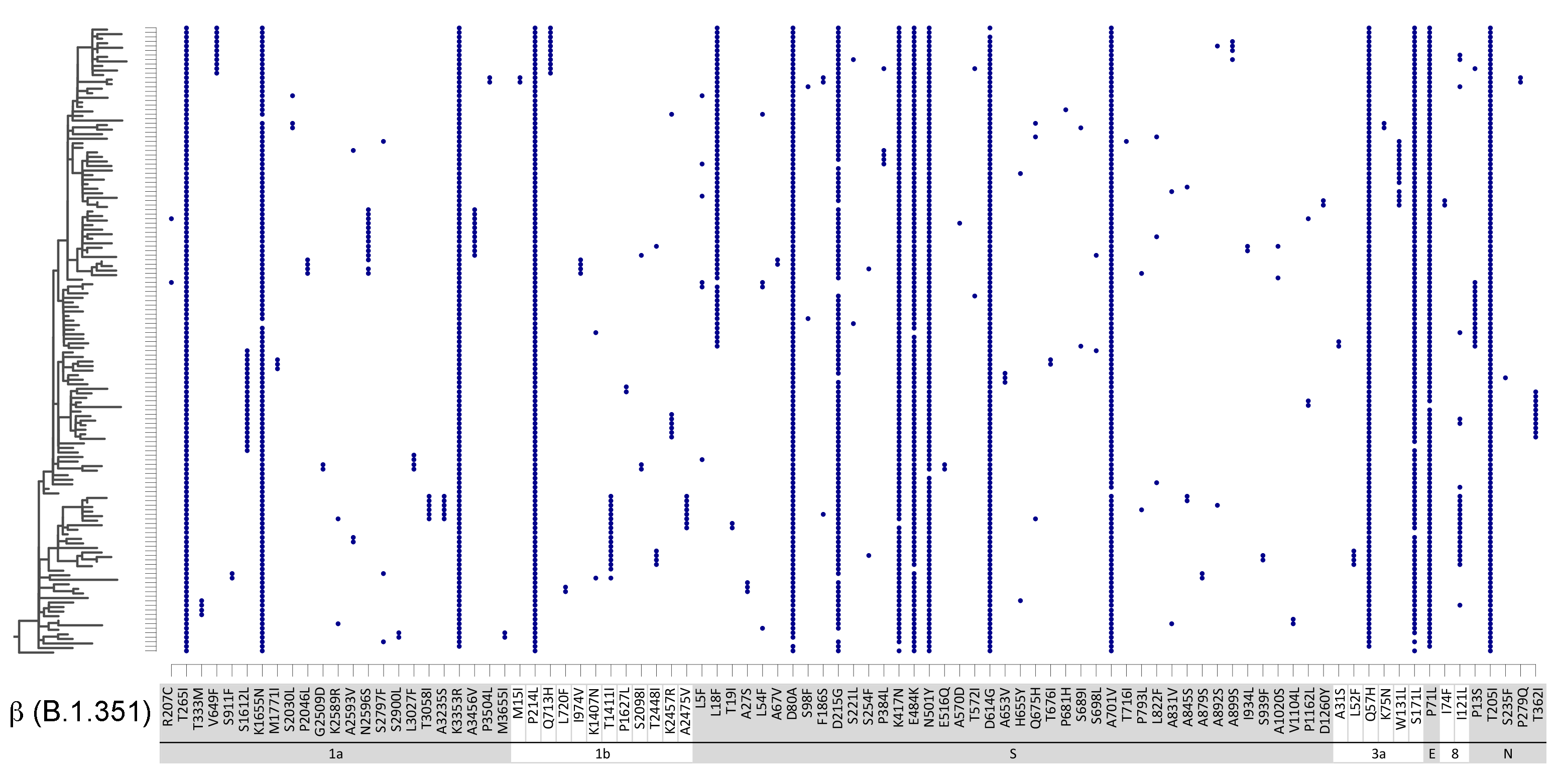

Supplement: FIG S2 [file mbio.02687-21-sf002.tif]

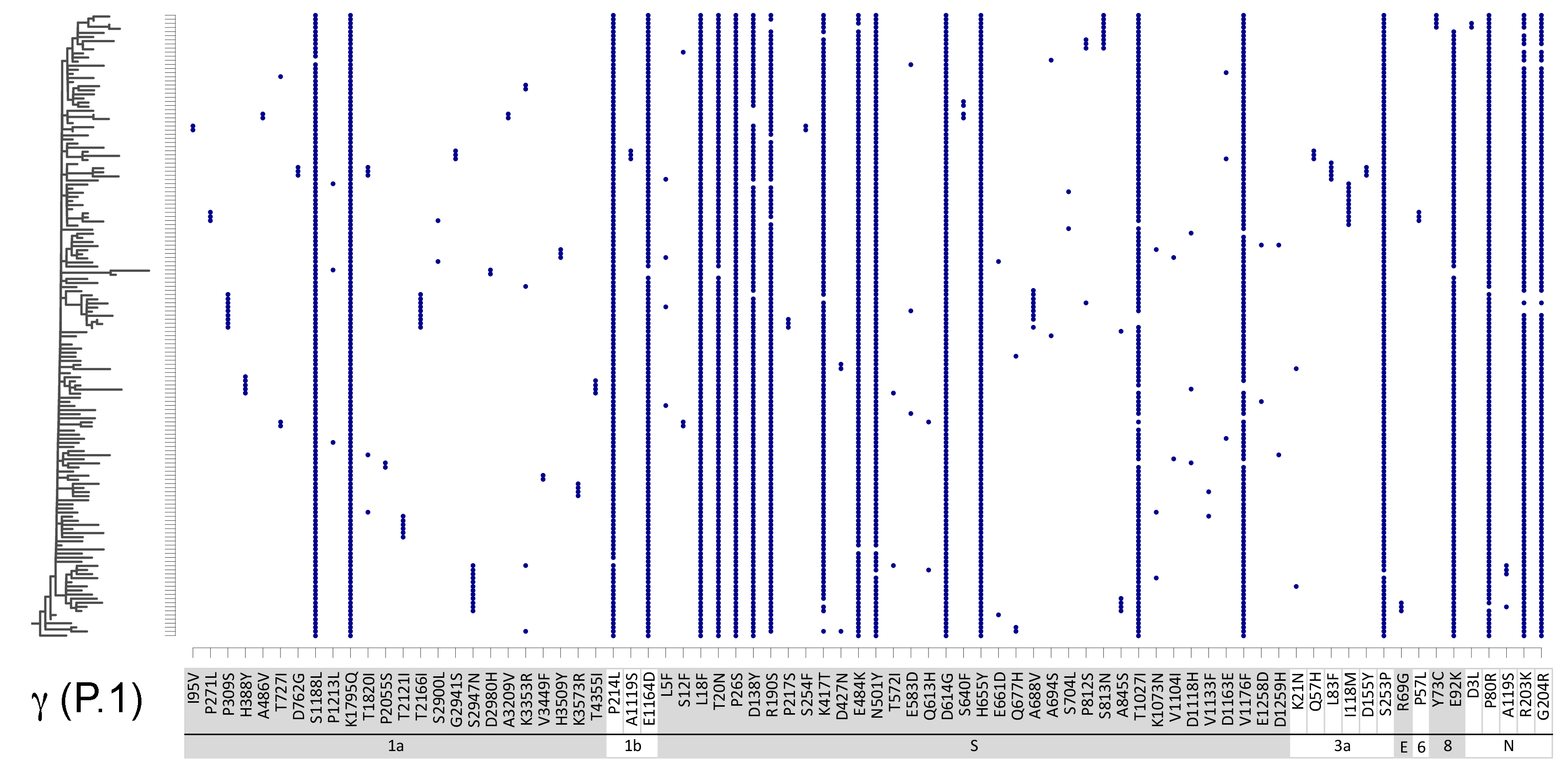

Supplement: FIG S3 [file mbio.02687-21-sf003.tif]

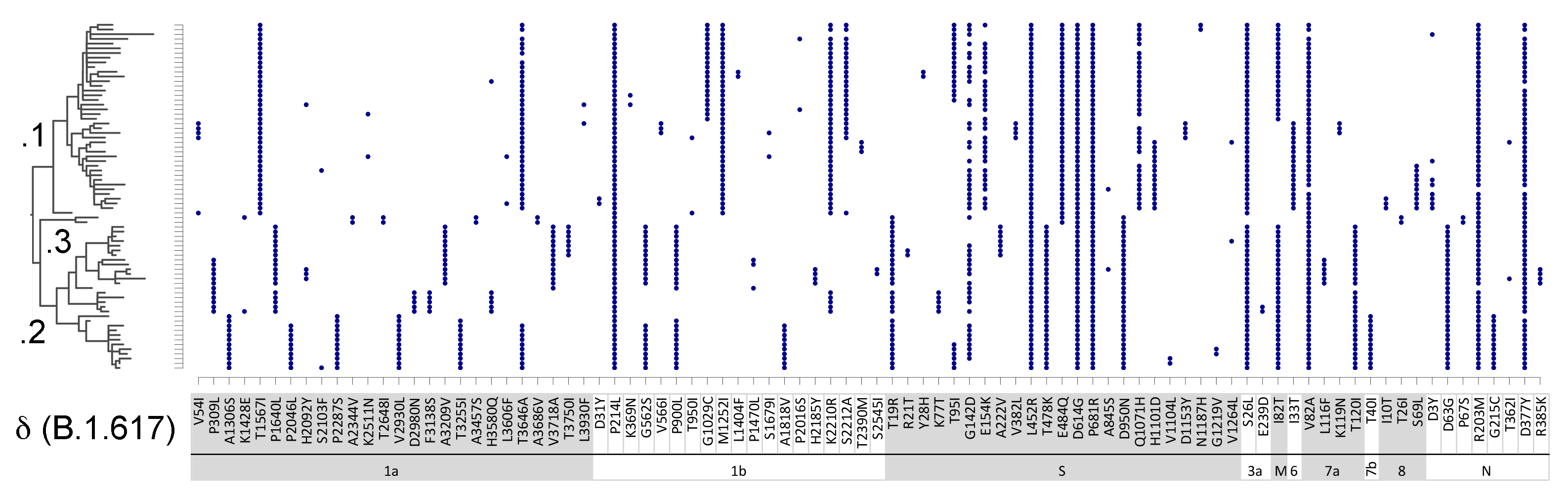

Supplement: FIG S4 [file mbio.02687-21-sf004.tif]

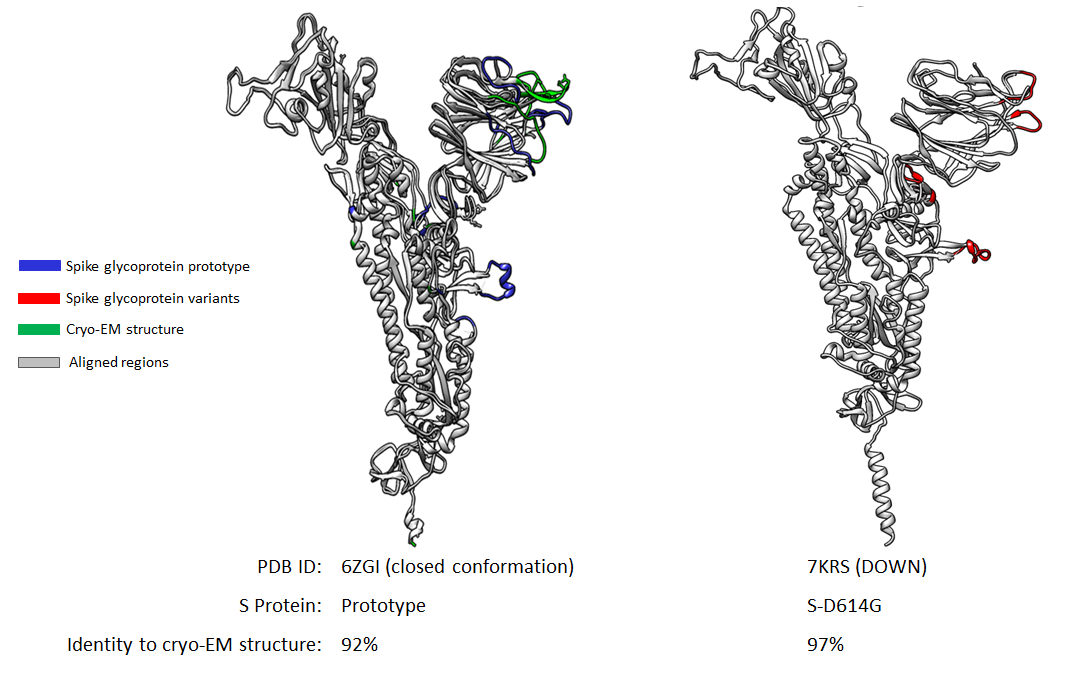

Supplement: FIG S5 [file mbio.02687-21-sf005.tif]
